# Highlighting the value of Alzheimer’s disease-focused registries: lessons learned from cancer surveillance

**DOI:** 10.3389/fragi.2023.1179275

**Published:** 2023-05-05

**Authors:** Margaret C. Miller, Rana Bayakly, Bernard G. Schreurs, Kimberly J. Flicker, Swann Arp Adams, Lucy A. Ingram, James W. Hardin, Matthew Lohman, Marvella E. Ford, Quentin McCollum, Audrey McCrary-Quarles, Oluwole Ariyo, Sue E. Levkoff, Daniela B. Friedman

**Affiliations:** ^1^ Department of Epidemiology and Biostatistics, Arnold School of Public Health, University of South Carolina, Columbia, SC, United States; ^2^ Office of the Study of Aging, University of South Carolina, Columbia, SC, United States; ^3^ Georgia Department of Public Health, Atlanta, GA, United States; ^4^ Department of Neuroscience, Rockefeller Neuroscience Institute, West Virginia University, Morgantown, WV, United States; ^5^ Department of Health Promotion, Education, and Behavior, Arnold School of Public Health, University of South Carolina, Columbia, SC, United States; ^6^ College of Nursing, University of South Carolina, Columbia, SC, United States; ^7^ Department of Public Health Sciences, College of Medicine, Medical University of South Carolina, Charleston, SC, United States; ^8^ Hollings Cancer Center, College of Medicine, Medical University of South Carolina, Charleston, SC, United States; ^9^ College of Social Work, University of South Carolina, Columbia, SC, United States; ^10^ Department of Health Sciences, South Carolina State University, Orangeburg, SC, United States; ^11^ Department of Biology, Allen University, Columbia, SC, United States

**Keywords:** public health, aging, cancer, disease surveillance, disease registries, Alzheimer’s disease and related dementias, data management, data infrastructure

## Abstract

Like cancer, Alzheimer’s disease and related dementias (ADRD) comprise a global health burden that can benefit tremendously from the power of disease registry data. With an aging population, the incidence, treatment, and mortality from ADRD is increasing and changing rapidly. In the same way that current cancer registries work toward prevention and control, so do ADRD registries. ADRD registries maintain a comprehensive and accurate registry of ADRD within their state, provide disease prevalence estimates to enable better planning for social and medical services, identify differences in disease prevalence among demographic groups, help those who care for individuals with ADRD, and foster research into risk factors for ADRD. ADRD registries offer a unique opportunity to conduct high-impact, scientifically rigorous research efficiently. As research on and development of ADRD treatments continue to be a priority, such registries can be powerful tools for conducting observational studies of the disease. This perspectives piece examines how established cancer registries can inform ADRD registries’ impact on public health surveillance, research, and intervention, and inform and engage policymakers.

## 1 Introduction: Registries as a powerful source of data surveillance and for enhancing public health

Evidence-based decisions in public health are often guided by the interpretation of surveillance data (Bauer, 2014). Surveillance is the ongoing systematic collection of health-related data that public health researchers aggregate, analyze, and disseminate for making informed public health practice decisions (Langmuir, 1963). *Public health surveillance—*as first coined by Thacker and Berkelman in 1988 and most recently refined in 2012 by the Centers for Disease Control and Prevention’s (CDC) surveillance working group, addresses a defined public health problem and is intended to reduce morbidity and mortality and improve population health (L. M. Lee and Thacker, 2011; Thacker et al., 2012; Thacker and Berkelman, 1988).

Registries are a powerful source of data surveillance (Declich and Carter, 1994) and may be classified as a product registry, health service registry, or disease (condition) registry (Gliklich et al., 2014). Disease registries are based on data from people who share a standard feature that defines the registry’s purpose. The information in disease registries is updated, predefined, systematic, and periodic, usually based on a geographically defined population (Donaldson, 1992). Disease incidence is reported to one of the three levels of registries, including local hospitals, central registries (hospitals/regions), and population-based registries (Rankin and Best, 2014). The four aims of a disease registry include the intention to: 1) improve patient care, 2) enhance public health, 3) advance medical knowledge, and 4) disseminate information (Rankin and Best, 2014).

Beyond being a representative source of de-identified data for a defined population (Gliklich et al., 2014), registries provide rich resources for observational studies (Hlatky et al., 1984), improving study design, process, and hypothesis testing (Porten et al., 2011; Gliklich et al., 2014). Linking this aggregation of complete, high-quality, timely data to other data collections like biobanks and randomized control trials (RCTs) has expanded population-based studies (Maudsley and Williams, 1999; Li et al., 2016; Hoskin et al., 2019; Karanatsios et al., 2020; Hoopes et al., 2021).

Using standard nomenclature (for disease etiologies, stages, and treatments) has allowed registry-based trials to compare studies in the real world with existing clinical care practices to determine real-world outcomes (Karanatsios et al., 2020). Involving the diverse perspectives of users, creators, and sources of data—cancer registrars, patients, caregivers, and providers—in creating and evaluating the registries provides critical perspectives for relevant, high-functioning, sustainable registries (Maudsley and Williams, 1999; Parkin, 2006; Bray and Parkin, 2009; Bray et al., 2014; Gliklich et al., 2014; MacIntyre and MacKay, 2018). Cancer registries, in particular, are regulated, linked, and share nomenclature across disease types, stages of diagnosis, and treatments (NAACCR, 2020), expanding the possibilities of innovative cancer prevention and control approaches to address the most common and rarer cancers competently, powerfully, cost-effectively, and compassionately across the continuum of cancer care (B. Lee et al., 2022; Mariotto et al., 2011; Piñeros et al., 2017; Ribisl et al., 2017; Wingo et al., 2005). This paper examines how established cancer registries can inform Alzheimer’s disease and related dementias (ADRD) registries’ impact on public health surveillance, research, and intervention.

## 2 History of the development of cancer registries

Investigators interested in establishing disease registries have much to learn from the most extensively developed disease registries in the United States (US)—cancer registries (Cromley and McLafferty, 2012, p. 93). In the case of cancer, the second leading cause of death worldwide, tracking characteristics of cases and the morphology of such a heterogenous disease through registries is well-established and essential to surveillance and cancer control programs worldwide (Parkin, 2006; Ferlay et al., 2021). The core purpose of a cancer registry is to estimate the burden of cancer with a focus on risk (incidence) based on the defined regional or national population. In the US, all states mandate cancer reporting (Coates et al., 2015). This state-required reporting, combined with federal collaboration with both the CDC and the National Cancer Institute (NCI), creates a vast and complex network of data sources that ultimately support the infrastructure for two national cancer registries, NCI’s Surveillance, Epidemiology, and End Results (SEER) and CDC’s National Program for Cancer Registries (NPCR).

In 1973, the SEER program established a coordinated system of cancer registries from the research of two pre-existing cancer surveys, the Third National Cancer Survey (Cutler and Young, 1975) and the End Results Program (Ederer, 1961). At that time, SEER included five states and two large metropolitan cities. Since then, it has collected quality data on patient demographics, tumor locations, morphology, and stage of diagnosis, as well as treatment and follow-up information for approximately 30% of the US population, encompassed by ten states and seven regions (Bray and Parkin, 2009; White et al., 2017). A key strength of SEER is its expandability and linkages with administrative data, including that from the National Death Index, Social Security Administration, Medicare, Medicaid, and state vital records departments. Linking to such complementary databases provides opportunities for researchers to identify disparities (Francoeur et al., 2022; Lawson et al., 2022), costs (Islami et al., 2022; Shih et al., 2022), risk reduction interventions (Hurwitz et al., 2022), and emerging trends (Chang et al., 2022; Shen et al., 2022), among many other findings. In 1992, through Public Law (PL 102–515), the US Congress created the Cancer Registries Act charging the CDC to form and fund the National Program for Cancer Registries (NPCR), incentivizing and standardizing state registries across all 50 states. The NPCR encompasses the remaining states and territories not included in the SEER database and overlapping areas, ultimately covering 96% of the US population. SEER and NPCR work closely with NAACCR (North American Association of Central Cancer Registries). NAACCR is the collaborative umbrella organization for North American cancer registries. It notably develops and promotes uniform data standards for cancer registration; provides education and training; certifies population-based registries; aggregates and publishes data from central cancer registries; and promotes cancer surveillance data from such systems as SEER and NPCR (NAACCR, 2016b). It combines registry information from Canada for even more power in cancer data aggregation (NAACCR, 2016a).

In South Carolina (SC), for example, the SC General Assembly passed the SC Central Cancer Registry Act in 1996, creating the South Carolina Central Cancer Registry (SCCCR), the state’s population-based cancer surveillance system. The SCCCR has consistently achieved Registry of Distinction and Gold Certification status with the NAACCR. The SC Department of Health and Environmental Control (DHEC) collects, processes, analyzes and publishes SC cancer incidence data which annually feed into the networks of the CDC and the NPCR (SCDHEC, 2022). With SCCCR keeping comprehensive and accurate records for surveillance, cancer researchers have identified: risk and prevention factors (Wagner et al., 2011; Tantamango-Bartley et al., 2013; Orlich et al., 2015; Fraser et al., 2020b; Babatunde et al., 2021), the timing of diagnosis-to-treatment and access to care (Virgo et al., 2010; Babatunde et al., 2022), and disparities in prevalence among different racial groups (Adams et al., 2006; Meyer et al., 2007; Adams et al., 2009; Babatunde et al., 2021; Thomas et al., 2021; Adams et al., 2022), geographic groups (Adams et al., 2006; Meyer et al., 2007; Georgantopoulos, 2018; Nicoli et al., 2019; Babatunde et al., 2021; Adams et al., 2022; Babatunde et al., 2022) and religious groups (Fraser et al., 2020a). Using the SCCCR, researchers have found trends suggesting efficient and effective treatment (Yen et al., 2006; Overton et al., 2013; Noxon and Bennett, 2015; Xirasagar et al., 2015) and intervention elements needed to address the person and community coping with cancer (Coker et al., 2006). When the SCCCR data gets aggregated into the national and multinational registries, NPCR and NAACCR, respectively, researchers can define the impacts of the more robust surveillance in a richer context (Ferlay et al., 2021; Zahnd et al., 2021).

## 3 Learning from cancer registries in the surveillance of Alzheimer's disease and related dementias

Like cancer, Alzheimer’s disease and related dementias (ADRD) represent an insidious global health burden that can significantly benefit from the power of accumulating disease registry data. With an aging population, the incidence, treatment, and mortality from ADRD are increasing and changing rapidly (Alzheimer’s and Dementia, 2023). In the same way that current cancer registries work toward control and prevention, so do ADRD registries. ADRD registries maintain a comprehensive and accurate registry of ADRD within their state, provide disease prevalence estimates to enable better planning for social and medical services, identify differences in disease prevalence among demographic groups, help those who care for individuals with ADRD, and foster research into risk factors for ADRD. Additionally, ADRD registries offer a unique opportunity to efficiently conduct high-impact, scientifically rigorous research without the burden of primary data collection. With the explosion of electronic health record systems mandated by our federal government, the efficiency of ADRD registries as a research resource through key data linkages could be expanded exponentially and strengthened through the establishment of these registries on a national level.

Given the nearly 50-year history available and the expansive scope across all 50 states, lessons learned from developing, implementing, and maintaining cancer registries can help decrease the time investment needed to develop, implement, and maintain such a system in ADRD. Perhaps even more importantly, identifying the weaknesses of our current cancer registry system is critical to creating a surveillance system that can surpass cancer registries in their utility. These established systems also inform us about mistakes that can be avoided with the hindsight offered by the cancer registry process. These may include the lack of a unified data collection system for all states and the creation of multiple national registries as opposed to one comprehensive registry. Finally, the vision of the future for cancer registry operations enables ADRD systems to strategically plan and begin early implementation of systems and processes that exceed our cancer registry system.

## 4 Formation and description of existing ADRD registries

Currently, there are three statewide ADRD registries in the US, all of which are geographically located in the southeast. While gaining attention, the registries are currently underutilized. The SC Alzheimer’s Disease Registry began in 1988. On 31 May 1990, Governor Carroll A. Campbell, Jr. signed a state law authorizing the Registry. This law (R653, H4924) amended Title 44, Code of Laws of South Carolina 1976, relating to health, by adding Chapter 36, establishing a voluntary Statewide Alzheimer’s Disease and Related Dementias Registry located within the School of Public Health at the University of South Carolina. The law has strict confidentiality requirements for data collection using existing sources. Still, it does allow Registry staff to contact the families and physicians of persons diagnosed with ADRD to collect relevant data and provide information about public and private healthcare resources and services available to them. It is maintained by the University of South Carolina’s Office for the Study of Aging with the support of the South Carolina Department of Health and Human Services and the Revenue and Fiscal Affairs Office. An annual report (65) with summary data is published in fulfillment of the requirement of the South Carolina Code of Law Section 44 36 10 and Section 44 36 50, which established the registry for the state and tasked the University of South Carolina’s Arnold School of Public Health Office for the Study of Aging with managing the registry data. Since 1 January 1988, the Registry has identified 340,921 cases of ADRD in South Carolina. Data from the SC Alzheimer’s Disease Registry is pulled from multiple sources, including in-patient hospitalizations, emergency room visits, long-term care evaluations, state health plans, Medicaid, Vital Records, Home Health, Community Mental Health Centers, Mental Health and Rehabilitation Clinics, and Program of All-inclusive Care for the Elderly (PACE).

Based on the model developed in SC, faculty of the West Virginia (WV) University School of Medicine, together with representatives of the WV Chapter of the Alzheimer’s Association, the Department of Health and Human Resources, the Blanchette Rockefeller Neurosciences Institute, and the South Carolina Alzheimer’s Disease and Related Dementias Registry formulated and proposed legislation that would establish a registry of people in West Virginia with AD and related dementias. This legislation was introduced to the West Virginia Legislature on 11 January 2006, as Senate Bill 112 by Senator Roman Prezioso, Chair of the Senate Health and Human Resources Committee, and sponsored by all the committee members. SB 112 passed on 11 March 2006, and became law on 11 June 2006, (WV Code §16-5R-7). Following the legislative process, procedural rules governing the type and manner of data collection for the West Virginia Alzheimer’s Disease Registry (WVADR) were written and went into effect on 27 December 2007 (CSR64-94). WVADR is an electronic population-based registry that collects demographic, diagnosis, and treatment information about ADRD and serves as an information repository for policy, planning, and research concerning ADRD. It is password-protected, encrypted, and located on servers in a secure facility.

During the 2013 Georgia legislative session, the Georgia General Assembly created the Georgia Alzheimer’s and Related Dementias (GARD) State Plan Task Force. The GARD Healthcare Research and Data Collection subcommittee found a paucity of data about ADRD in Georgia, yet no central repository existed for these data. Thus, it created a barrier to estimating accurate ADRD prevalence rates in Georgia to inform planning, research, and reporting efforts. The task force created a State Alzheimer’s Disease Plan, including recommendations to collect statewide data to inform the evaluation and care infrastructure. A key recommendation was establishing a statewide ADRD registry to provide accurate, current data to address these urgent needs. During the 2014 Georgia Legislative Session, legislation to establish an ADRD registry within the Georgia Department of Public Health (DPH) (HB 966) was introduced and subsequently passed (OCGA 31-2a-17). The Georgia Department of Public Health (DPH) was identified as a prime coordinator of stakeholders and partners in the registry planning and development effort.

In 2019 the OCGA 31-2a-17 further defined the purpose, procedures, rules and regulations, and data confidentiality of the ADRD registry. Based on Georgia Law, the purpose of the ADRD registry is to assist in the development of public policy and planning, provide a central database of individuals with ADRD, establish procedures and promulgate rules and regulations for establishing and operating the registry. According to the Georgia Law, such procedures, rules, and regulations were intended to provide for 1) collecting and evaluating data regarding the prevalence of ADRD in Georgia, including who reports the data to the registry; 2) determining what information shall be maintained in the registry and the length of time such data shall be available; 3) sharing of data for policy planning purposes; 4) disclosing non-identifying data to support ADRD research; and 5) information about public and private resources. The methodology by which families and physicians of persons who are reported to the registry are contacted to gather additional data is also provided. The law stated that the collected ADRD registry data should be confidential. All persons to whom the data are released should maintain patient confidentiality under the requirements of 42 USC Section 1301, et seq., and PL 104–191, the federal Health Insurance Portability and Accountability Act of 1996.

## 5 Current and potential impact of ADRD registries on research, workforce, education, and policy

In addition to research on the statewide prevalence of ADRD (Office for the Study of Aging, 2022) and prevalence in special populations (Miller et al., 2023), ADRD registries have been used to investigate potential risk factors for the disease (Miller et al., 2019) and impacts on those who provide care for individuals with ADRD (Porter et al., 2016; Carpenter et al., 2020; Alhasan et al., 2021). With continued advancement in research on ADRD treatment and prevention in the scientific community and mass media, having such ADRD registries for observational studies with such a large resource of patient records will also be extremely important for understanding the real-world impact of emerging interventions.

While the scientific community is becoming increasingly diverse, there is still an underrepresentation of individuals from minority groups pursuing research careers in aging and ADRD despite the increased risk of ADRD within those groups (Johnson et al., 2022). Research centers encourage using ADRD registry data to encourage population-level studies to decrease ADRD-related health disparities. Currently, the Carolina Center on Alzheimer’s Disease and Minority Research (CCADMR; P30 AG059294), a National Institute on Aging (NIA)-funded center dedicated to increasing the capacity of underrepresented and minority (URM) scholars, is working to advance the science of ADRD research focused on population health and determinants of ADRD disparities through research education in population-based, secondary data analysis, interdisciplinary co-mentoring teams, well-established strategies for recruitment of AD-RCMAR Scientists, and education on Health Disparities and Minority Aging Research. The SC Alzheimer’s Disease Registry is a key data source for CCADMR Scientists (Ingram et al., 2021; Johnson et al., 2022).

Additionally, a 5-year NIA-funded grant (R13 AG074603) offers an annual virtual conference on how to use data from all three statewide registries for studying ADRD disparities. Furthermore, it involves a follow-up High-Impact Alzheimer’s Disease Registry Workshop for Scholars of Color—a 1-day workshop for mentored URM scientists interested in developing a research project using SC Alzheimer’s Disease Registry data with the support of a mentor. The workshop aims to introduce scholars to available registry data opportunities to brainstorm project ideas, network with other scholars, and connect with a group of senior mentors whom they meet with over the next full year. No prior research experience with registries is required.

ADRD registry data can also inform education, including the nationally registered Dementia Dialogues program for caregivers of persons who exhibit signs and symptoms of ADRD (Byers et al., 2022). The six-module program, which presents data from the SC Alzheimer’s Disease Registry, has at its mission to provide the most current and practical evidence-based information about how to care for people living with ADRD. The target audience includes formal and informal caregivers and other community members interested in learning more about ADRD caregiving. Communities across the state, especially those with the highest ADRD rates based on Registry data, are engaged with this evidence-informed education and programming.

Finally, ADRD registries have the potential to inform policy. For example, county-level fact sheets are developed annually for the Alzheimer’s Association SC Chapter to present to legislators and community partners, and state agencies to promote awareness of ADRD across urban and rural counties of the state with the goals of improving ADRD education, access to care, and advocate for additional funding for patients and caregivers. A similar county-level effort in WV by the WV Alzheimer’s Disease Registry and Alzheimer’s Association WV Chapter volunteers led to a series of datasets sorted by legislative district and used to raise awareness and generate support for Alzheimer’s disease among WV legislators. One tangible result was the passage of legislation (Senate Bill 570) to provide training to police and fire personnel engaging with people with Alzheimer’s disease. A second bill (Senate Bill 526) was passed in 2023 to increase ADRD training for healthcare providers.

## 6 Concluding remarks: Lessons learned for ADRD registries moving forward

ADRD registries are impactful in many areas, including surveillance, research, and policy. There is also enormous potential for enhancement and expansion. As highlighted in Table 1, ADRD registries currently lack consistent and standard reporting of disease staging (e.g., preclinical, early-late), outcomes of treatment, imaging (e.g., PET, fMRI), biomarker ascertainment (e.g., pathogenic proteins, markers of synaptic dysfunction, and markers of inflammation in the blood), genetic testing (e.g., APOE gene), long-term follow-up information (e.g., preclinical/early stage through late stage), quality of life measures, or caregiver/family demographic and health information. Some similar information is captured in cancer registries and the availability of these data has led to groundbreaking discoveries over the past few decades.

**TABLE 1:** Comparing available data across registries.

	Cancer registry	Alzheimer’s disease registry
Demographic Information	yes	yes
Family/Caregiver Demographic and Health information	no	no
Disease Type	yes	yes
Age at Diagnosis	yes	yes
Year of Diagnosis	yes	yes
Geographic Location at Diagnosis	yes	yes¥
Staging of the Disease at Diagnosis	yes	no
Plan of Treatment		
*First course of treatment*	yes	**
*Outcome of treatment*	no	no
Medication use	no	**
Imaging data	limited*	no
Biomarkers	some↑-limited*	no
Medical procedures	limited*	**
Genetic testing	some↑-limited*	no
Hospitalization information	no	**
Death Data		
*Vital Status*	yes	yes
*Date of Death*	yes	* (year only)
*Underlying cause of death*	yes¥	yes
*Other information from death certificate*	no	**
Quality of life measures	no	no
Long-term follow-up data	no	no

↑- Indicates that it is not a required data element, but some registries will independently capture this information.

*limited-indicates that registry will capture some data related to this outcome, but do not collect a comprehensive treatment record.

**limited data is available through linkages to other data sources after receiving proper permissions.

¥—is a required data element, but privacy restrictions can limit its availability for research.

As the ADRD registry system is still in the development stage, there are several pitfalls to avoid and lessons learned from the development of cancer registries which, if considered early in the process, have the potential to benefit the ADRD system.

• One of the greatest barriers to cancer research is the lack of a single unified system. The infrastructure allows for research to be conducted on the entire SEER database, however, research using state data from NAACCR registries has to be conducted on a state-by-state basis. Thus, having a truly national cohort of cancer survivors for research is impossible.
• Furthermore, states that are members of the NAACCR system operate autonomously and can opt in or out of research studies that want to utilize their data, even though all cancer registries are funded at the federal level. As ADRD registries develop, it would be wise to keep them unified under a single system.
• Another key point is to establish a way to systematically link ADRD registries to multiple other data sources, including claims data. Similar to how the NAACCR brings interdisciplinary teams together to set cancer definitions, data dictionaries, and data performance measures, bringing experts who are knowledgeable in information technology and data sharing, perhaps including, but not limited to public health, will be critical to have at the table during all development phases of ADRD registries to ensure that we build a system with the flexibility to evolve over time and stay relevant with health data sources. A key strength of SEER is its expandability and linkages with administrative data. Regarding ADRD registries, once expanded and developed, data linkages such as SEER-Medicare would also add treatment and cost for the Medicare population which would include most patients with ADRD. For non-Medicare patients, SEER specifically has linked cancer registry data with administrative data and pharmacy data to enhance and expand the use of cancer registry data. This type of expansion would enhance future risk reduction and treatment interventions for ADRD.
• Finally, utilizing the fullest extent of existing protocols and processes from the NAACCR data standard will be essential to allow ADRD registries to become operational efficiently while allowing experts and key stakeholders to focus on issues that may be unique to ADRD.

## Data Availability

The original contributions presented in the study are included in the article/Supplementary Material, further inquiries can be directed to the corresponding author.
